# Integration of Railway Bridge Structural Health Monitoring into the Internet of Things with a Digital Twin: A Case Study

**DOI:** 10.3390/s24072115

**Published:** 2024-03-26

**Authors:** Alberto Armijo, Diego Zamora-Sánchez

**Affiliations:** TECNALIA, Basque Research and Technology Alliance (BRTA), Astondo Bidea, Edificio 700, 48160 Derio, Spain; diego.zamora@tecnalia.com

**Keywords:** structural health monitoring (SHM), railway bridges, digital twin (DT), machine learning (ML), MLOps, low-cost MEMS accelerometers, vibration-based monitoring, wireless sensor networks (WSNs), hybrid computing, building information modeling (BIM)

## Abstract

Structural health monitoring (SHM) is critical for ensuring the safety of infrastructure such as bridges. This article presents a digital twin solution for the SHM of railway bridges using low-cost wireless accelerometers and machine learning (ML). The system architecture combines on-premises edge computing and cloud analytics to enable efficient real-time monitoring and complete storage of relevant time-history datasets. After train crossings, the accelerometers stream raw vibration data, which are processed in the frequency domain and analyzed using machine learning to detect anomalies that indicate potential structural issues. The digital twin approach is demonstrated on an in-service railway bridge for which vibration data were collected over two years under normal operating conditions. By learning allowable ranges for vibration patterns, the digital twin model identifies abnormal spectral peaks that indicate potential changes in structural integrity. The long-term pilot proves that this affordable SHM system can provide automated and real-time warnings of bridge damage and also supports the use of in-house-designed sensors with lower cost and edge computing capabilities such as those used in the demonstration. The successful on-premises–cloud hybrid implementation provides a cost effective and scalable model for expanding monitoring to thousands of railway bridges, democratizing SHM to improve safety by avoiding catastrophic failures.

## 1. Introduction

Railway bridges are indispensable components of global transportation networks and play a crucial role in facilitating the seamless movement of goods and passengers. Ensuring their structural integrity is not just a matter of maintenance but a critical safety imperative. Traditional methods of inspecting this vital infrastructure through visual surveys are not only resource-intensive but also sporadic and disruptive to daily operations. Recognizing these challenges, this article introduces an innovative approach to structural health monitoring (SHM) of railway bridges, integrating the principles of the Internet of Things (IoT) with a digital twin framework. This solution employs low-cost wireless sensors and leverages advanced machine learning algorithms to automate the monitoring process, offering real-time insights into the structural health of railway bridges.

Section 1 provides insightful background on railway bridges and highlights the importance of SHM for these critical assets. It also summarizes previous research efforts that independently explored components like low-cost sensing, energy harvesting, digital twins, and machine learning for vibration-based SHM of bridges. Finally, the section introduces the proposed digital twin solution for SHM of railway bridges.

Section 2 outlines the key objectives and performance metrics of our study, including achieving high classification accuracy for detecting abnormal vibration patterns using cost-effective sensors, facilitating widespread deployment through a hybrid computing architecture, and reducing installation and maintenance complexity.

Section 3 describes the system architecture, detailing the physical components, like wireless accelerometers and gateways, as well as the digital components, comprising on-premises middleware for real-time processing and a cloud platform for data storage, batch analytics, and machine learning model management.

Section 4 presents our experimental methodology, introducing a comprehensive seven-step data-driven process as a generic framework for SHM (Section 4.1). This replicable methodology covers bridge characterization, sensor deployment, data preprocessing, unsupervised learning of normal vibration patterns, model calibration using finite element analysis, generation of synthetic damage data, supervised model training, and continuous learning through periodic retraining. Section 4.2 discusses the specific application of this methodology to a pilot railway bridge, including an overview of the field deployment, sample acceleration data, and insights into how the digital twin detects structural changes.

Section 5 discusses the results, summarizing the digital twin approach’s capabilities, comparing its performance to the existing literature, and quantifying the demonstrated benefits of low-cost and rapid damage detection enabled by the hybrid edge–cloud architecture.

Finally, Section 6 concludes the article, highlighting the successful real-world validation and outlining future research directions, such as expanding to broader production deployments, improving model fidelity through advanced simulations, and exploring applications for monitoring other infrastructure like wind turbines.

By integrating IoT sensing, edge–cloud computing, digital twins, and machine learning, this work establishes a pragmatic and scalable framework for continuous, automated monitoring of railway bridge structural integrity. The pilot implementation not only demonstrates the system’s reliability, accuracy, and cost-effectiveness but also marks a significant step toward ensuring safer and more resilient transportation infrastructure through data-driven SHM.

### 1.1. Brief Background of Railway Bridges and Importance of Structural Health Monitoring

The transport system—in particular, the railway system—is essential to our daily lives, and plays a fundamental role in the social and economic development of a region and a country [1]. The transport networks in the Basque Country (Spain) and in Europe are highly developed but face a growing problem of aging as, for example, 35% of European railroad bridges are more than 100 years old [2]. Also, many of these bridges, especially the relatively old ones, are being pushed beyond their designed physical capacities due to the increased speed, axle load, length, and travel frequency of new trains and transportation needs [3,4,5,6].

Climate change introduces uncertainties, since previously unseen climatic conditions and phenomena tend to occur more frequently and unexpectedly, making these structures particularly vulnerable [7]. In addition, bridges, given their relatively low level of structural redundancy, are generally at risk of collapse in the event of significant damage caused by deterioration, resulting in serious economic losses, interruptions in the normal course of people’s lives, and, in the worst case, irreparable tragedies.

Recently, there have been several major bridge collapses around the world, including the Morandi Bridge failure in Genoa in 2018 [8] and the Nanfang’ao Bridge collapse in Yilan County in Taiwan in 2019, among others [9]. These incidents show the potential dangers when critical structural damage goes undetected. As a result, frequent and proactive monitoring of bridge structural health is essential to avoid such catastrophic failures. Minor issues like cracking or loose connections can gradually worsen over time if not spotted early.

Taking a broad definition, structural health monitoring (SHM) is the process of assessing the state of health of a structure based on data from instrumentation installed on the structure [10,11]. It is a process that can be divided into different phases depending on the criterion: some do it in three, such as in [12], although we distinguish four, since management or decision making, facilitated by contextualized access to the data, is considered within the process in the present work:

- Instrumentation: Set of sensors and data acquisition systems that collect the physical structural parameters to be monitored and analyzed.
- Monitoring: Remote data transmission and web publication.
- Analysis: Set of techniques to convert the data into characteristic variables or parameters to understand the structural behavior and implement systems to evaluate and detect structural damage.
- Management: Decision-making aid for action and maintenance that involves making this real-time information available to the right people at the right time and within a realistic geometrical contextualization.

Due to their high costs, installing sophisticated structural monitoring systems in bridges is uncommon, so their application is restricted to singular bridges with special relevance. However, due to the obvious improvements that their implementation leads to, their use for bridges in general would be justified by developing an optimized solution for alerts and diagnostic support at a relatively reduced cost, such as the integrated system presented in this work, which employs advanced analysis techniques and IoT technology.

On the other hand, traditional on-site inspection methods are costly, since they require the travel of one or more technicians to each bridge to carry out the survey in order to evaluate the condition of the structure and, finally, to report it, and it may be necessary, particularly in the case of railway bridges, to interrupt or reduce the service to carry out some inspections. In this regard, if an automated alert system were commonly implemented to control the state of health of the structure, inspections could be optimized in number and form, and improved safety could be achieved in real-time instead of periodically. However, the main inspections and load tests required by the regulations in force in each case will always have to be carried out according to the scheduled preventive maintenance plans of the bridge structure.

### 1.2. Related Research Summary

This article builds upon and extends previous studies and has independently explored components like low-cost sensing, energy harvesting, digital twins, and machine learning for vibration-based structural health monitoring (SHM) of bridges.

Vibration-based monitoring is a widely extended practice that tracks the dynamic response of a structure [13,14]. Continuous vibration monitoring provides a data-driven method to detect subtle changes in bridge integrity. The implementation of an SHM system combining structural monitoring and machine learning [15,16,17,18] through an interactive digital twin (DT) that allows contextualization of bridge geometry and sensor position can be a key tool for making decisions to avoid infrastructure disasters. As defined in [19,20] a DT is a technology that enables the virtual representation of a physical system and its associated environment and processes. The system is continuously updated by exchanging information between the virtual and physical worlds. The DT integrates sensor data through a network of sensors from the physical asset to mirror its status, condition, and behavior in real-time. Recently, the DT concept has gained popularity and broad acceptance across industries and is being consolidated as a cornerstone of the Industry 4.0 paradigm. While manufacturing and naval sectors have developed many DT use cases, adoption in civil and structural engineering domain has lagged behind. Since 2018, the approach has been receiving more research attention in this domain, driven by the proliferation of the Industrial Internet of Things (IIoT) and market pull factors, such as the need for monitoring aging structures under changing use patterns and uncertain climatic conditions [20]. However, the integration of real-time sensor data with advanced simulation and AI algorithms remains an open challenge to realize the full benefits of digital twins for smarter and safer infrastructure management.

The article [21] proposes minimal information data modeling (MID) using low-cost (equipment costs around EUR 400) and easy-to-install sensors, which is also one of the key aspects of our proposal. In this sense, the novel technique presented in the aforementioned article offers high accuracy with low-range sensors and damage detection sensitivity down to 0.01 Hz frequency shifts, whereas in our approach, we rely on clustering that addresses the variability in identification due to multiple factors. This technique could also be suitable to be implemented in the digital twin platform presented in this research through cloud processing of the obtained records.

The article [22] proposes an automated framework to classify anomalies (i.e., drift, distortion, outlier, anomaly, bias, etc.) in the time domain and assess the current state of the structure, while in our approach, we work in the frequency domain, proposing clustering for anomaly detection after the identification of frequencies in free vibration, which also allows us to identify and discard faulty measurements. If a permanent discard were to occur, a no-data alert would also be issued, and we would have the corresponding signals in the time domain to perform a more detailed study or even accommodate algorithms such as the one proposed in the aforementioned paper since our digital twin system or architecture is flexible enough to include other algorithms and is capable of receiving and storing data in the time domain with high sampling frequencies.

The work in [23] offers a new approach to damage identification based on the extraction of continuous time series of autoregressive (AR) coefficients from deformation measurements on a railway bridge, but it is based on fiber optic technology, i.e., expensive instrumentation, while the application of the present work is based on low-cost accelerometry after the passage of a train. In any case, the core of our work is a digital twin system with a middleware component that can be readily adapted to other types of sensors with any physical magnitude provided that communication can be implemented at least from a PC connected in situ, while high-level algorithms, such as the one proposed in the work cited above, could be included in our processing layer.

The article [24] offers a bibliographic review of how energy harvesting technologies can provide sustainable power sources for wireless sensor networks (WSNs) deployed on bridges. The SHM systems implemented on bridges are mostly based on WSN. Solar, thermal, wind, and vibrational energy harvesting are all examined as ways to overcome the limitations of battery-operated sensor platforms. However, [24] was confined to an energy perspective and did not investigate how the data from such sensors could be utilized for automated SHM powered by simulations and analytics.

The work in [25] discusses that MEMS sensors are miniature in size and have lower cost and weight than conventional wired alternatives. These advantages make MEMS sensors better suited for permanent installation over many years of continuous infrastructure monitoring. The article also provides a bibliographic review regarding commonly used machine learning techniques, both classical and deep learning methods, for bridge structural health analysis using sensor data. But it identified high computational costs and model performance as limitations to practical cloud-based implementations for large-scale infrastructure monitoring. Edge computing is suggested as a potential solution, but it is not implemented.

The article [26] provides a review of machine learning algorithms that have been successfully applied in SHM, specifically in the domains of vision-based and vibration-based SHM. In this regard, this paper leverages a vibration-based approach. However, other, more advanced AI powered deep learning algorithms could be implemented using our demonstrated workflow.

### 1.3. A Digital Twin for SHM in Railway Bridges

The present work advances the state-of-the-art by combining the vital concepts from prior work, such as low-cost sensing, energy harvesting, and machine learning, into an integrated on-premises–cloud digital twin architecture with a successful real-world implementation. It makes several key contributions beyond these preceding studies. First, it demonstrates how minimal low-cost sensing can be integrated into a complete digital twin architecture: advancing from pure data science [21] to a production SHM system. In addition, our approach offers the added benefit of building information modeling (BIM) [27] contextualization of sensors and measurements within an architecture adaptable to other algorithms and sensors. Indeed, for this study, we designed and developed our own low-cost devices with edge computing capabilities compatible with the digital twin system. However, we prefer to present this digital twin as a system compliant with any sensor and any machine learning algorithm as well as adaptable to all types of structural configurations thanks to the geometric BIM contextualization of the bridge geometry and the instrumentation. Secondly, it implements sustainable solar energy harvesting in the full SHM solution, building on the potential shown in [24]. And thirdly, it delivers a hybrid edge–cloud machine learning pipeline to make large-scale analytics financially feasible, addressing the barriers called out in [25,26].

Specifically, our digital twin integrates inexpensive IoT acceleration sensors (equipment costs around EUR 300) with MQTT [28] connectivity, on-premises fog computing, cloud big data and machine learning services, and a visualization application. Compared to [21], the sensor data are augmented by their contextual placement within a digital twin model of the bridge for enhanced structural insights, with particular potential for monitoring local variables (such as strain) through the use of other types of sensors beyond accelerometers. This article applies the digital twin concept conforming to the definition given in [20] and comprises the next features:

- Simulation: SHM + IoT + BIM;
- Learning: AI;
- Management: DS (provides decision support).

The article [29] presents a pre-trained network with synthetic data, i.e., supervised with finite element models, which, based on deep learning techniques, could provide a fast response for damage identification if integrated with real-time monitoring, and which would be computationally suitable to be included in the digital twin system we present, thus extending its simulation performance for model inclusion (adding the MOD and DIAG function in the simulation capabilities according to the structural digital twin conceptualization presented in [20]).

Moreover, powering the wireless sensors using solar energy harvesting realizes a self-contained system, enabling the sustainable sensor networks envisioned by [24]. And by leveraging both real-time on-premises edge processing and cloud machine learning, our solution overcomes the prior constraints around computational costs described in [25,26], demonstrating affordable analytics scaling.

To handle the high-throughput vibration data generated by the accelerometers, the digital components of the structural health monitoring system were deployed in a hybrid on-premises and cloud architecture [30]. For real-time data ingestion and analysis, on-premises middleware was implemented. This on-premises system ingests the 500 Hz. sampled data streamed from the accelerometers on the bridges via MQTT and runs real-time machine learning algorithms to detect anomalies in the vibration patterns. To supplement this real-time analysis, the historical vibration data are also regularly forwarded to a cloud platform for longer-term storage and batch analysis. Storing and processing the entire high-frequency vibration dataset solely in the cloud would be prohibitively expensive due to large data volumes. By leveraging an on-premises system for time-critical analytics combined with cloud storage and batch processing, the railway operator can cost-effectively monitor the health of its bridges in real-time while also building up a knowledge base of historical structural dynamics data.

## 2. Objectives and Metrics

The aim of this work is to demonstrate and evaluate the capabilities of the proposed hybrid digital twin solution for short-span railway bridge structural health monitoring in terms of accuracy, cost-effectiveness, and ease of deployment compared to state-of-the-art methods. Specific objectives are to:

- Achieve ≥95% classification accuracy for detecting abnormal vibration patterns that indicate potential structural issues using only low-cost wireless sensors;
- Enable scalable large-scale monitoring across thousands of bridges through a cloud and on-premises edge computing architecture costing 80% less than traditional wired sensor networks;
- Reduce installation and maintenance complexity by over 50% compared to typical SHM systems by utilizing self-contained wireless sensors with battery/solar power.

Performance in these areas will quantify and demonstrate the improvements of the digital twin approach over current SHM practices in rail infrastructure monitoring in terms of precision, affordability, practicality, and democratization. The quantification is carried out through showcasing a fast, reliable, and cost-effective remote damage detection system for bridge structures that integrates the measured data and the alerts generated for the bridge into the Internet of Things (IoT). The system aims to move from a reactive to a proactive approach in bridge maintenance, replacing basic inspections with an automated process, to take a first step towards the smartization of this infrastructure and its management as Industry 4.0 [31] assets in a generalized way.

A pilot case of the system was implemented in a real environment for a railway bridge in the Basque Country. For this purpose, following a vibration signal data type approach and the application of a wireless sensor network (WSN) platform [24], an optimized sensor plan was developed for the structure, and the measurement information was remotely processed, enhancing its usability through its synthesized visualization on dashboards accessible from a geometric model in the cloud.

One major contribution of our present work is the deployment and evaluation of the digital twin capabilities in an operational context on a real railway bridge, which also proves the viability of AI-powered digital twins using low-cost wireless IoT sensors. For structural health monitoring of infrastructure like this bridge, the digital twin is powered by machine learning algorithms instead of traditional physics-based simulations. Data-driven approaches, e.g., [32], are widely used in SHM in both the time and frequency domains, but they are not usually based on low-cost IoT sensors leveraging the clustering-based approach of this work. This approach is particularly well aligned to work in real-time using Eigenfrequencies with uncertainty in their identification (not only environmental and operational but also due to sensor and measurement limitations). We first train the digital twin, as is the common practice in unsupervised data-based approaches, on vibration data collected from the bridge under known normal conditions. This allows the machine learning model to learn the patterns of vibration that correspond to normal structural dynamics. The trained digital twin model is then connected to real-time vibration data streamed from accelerometers on the actual bridge. By analyzing these vibrations using its trained machine learning algorithms, the digital twin can detect anomalies that deviate from the learned ”normal” patterns. These anomalies may indicate potential structural problems not discoverable through visual inspection.

Most of the traditional methods of operational modal analysis, or modal identification without input measurement, work with high-sensitivity and high-price accelerometers, such as the force-balance type, but in this case, we work with low-cost MEMS sensors, as the only valid measurable output is the one produced by the passage of the train, so that the free vibration after the exit of the train from the structure is a signal that contains only the natural frequencies of the bridge.

The digital twin model can be re-trained over time as more sensor data are collected to improve its accuracy. The machine learning approach provides a data-driven way to monitor bridge health without relying on complex physics simulations. By detecting vibration anomalies, the digital twin can provide early warning of damage so that repairs can be made before catastrophic failure occurs.

This work establishes a replicable and cost-effective methodology for real-time railway bridge monitoring that can be extended to large infrastructure networks. The approach demonstrates reliable high-frequency data collection using MQTT communication between low-cost sensors and cloud platforms. Compatibility with commercial off-the-shelf acquisition modules enables flexible adoption with existing monitoring hardware.

A hybrid on-premise and cloud architecture processes the high-volume sensor streams using open-source tools for edge analytics and cloud machine learning. Mosquitto, Node-RED, and time-series databases handle real-time needs, while cloud services provide scalable data storage, batch processing, and model management. The architecture is sensor-agnostic and adaptable to new data sources.

Additionally, a realistic digital twin integrates the real-time sensor data with bridge geometry models and AI-generated health insights for enhanced situational awareness. Interactive dashboards connect the physical infrastructure state with digital monitoring outputs.

Overall, this pilot study proves the real-world viability of transitioning from costly manual inspections to continuously automated AI-powered infrastructure health monitoring. By demonstrating a pragmatic digital twin system architecture using affordable off-the-shelf components, this work enables scalable structural monitoring to improve railway operations, maintenance planning, and passenger safety.

## 3. System Architecture Description

### 3.1. High-Level Overview

#### 3.1.1. Physical Components: Accelerometers Installed on Bridge and Local Gateway

The monitoring of structures is generally carried out only on bridges of special importance or in known poor condition and consist of extensive sensor networks with high unit cost. In this use case, although the developed system is compatible with any type of sensor (with or without a separate data acquisition system or a personal computer), low-cost wireless IoT sensors have been integrated, allowing high data acquisition rates and easy installation and communication through a 4G gateway located on site, and the entire system is energy self-sufficient through the use of solar panels.

**Figure 1:**
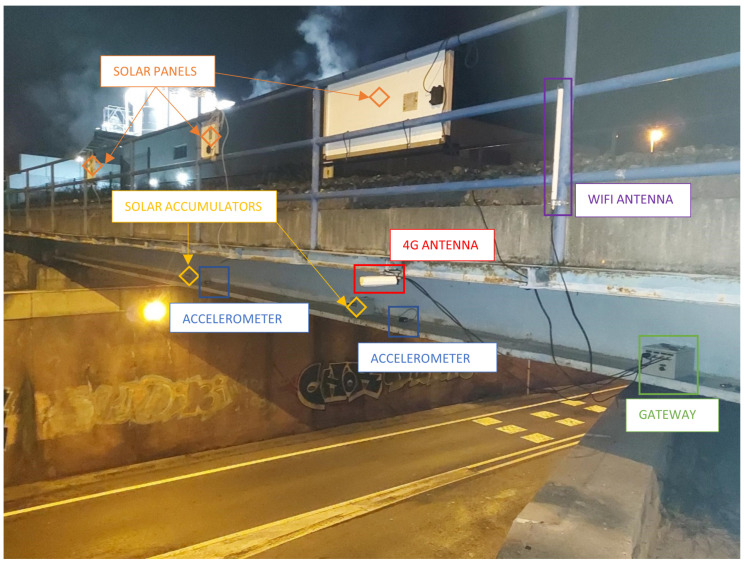
Sensor and communication layer, including a local WiFi network generated by the gateway and an MQTT publishing broker.

For the sensor and communications layer, as observed in Figure 1, communication of the sensors with the gateway was integrated through a local WiFi network generated by the gateway. With fewer cables than a conventional non-IoT system, the installation is faster, cleaner, and safer [33,34]. The MQTT protocol was used for sending data with a 3G network or higher, which allows high sampling transmission (500 Hz during most of the measurements taken). This allows for communication of the amount of data needed for a remote evaluation of this type of structure. Classical IoT protocols such as LoRa or SigFox (0G) are insufficient for civil engineering if data are not preprocessed by the sensor hardware through edge computing before being sent [35,36,37]. This preprocessing, which is complex and generates dependency on the referred hardware, may not be available or valid in every situation. Finally, we configured triggers for data capture activation at the initial pulse signal of a train’s crossing, which is the event of interest, thus saving data and energy consumption.

Among other aspects, transport infrastructure is a particular case in terms of:

- The typology and materials used as well as construction uncertainties;
- The failure modes to be considered: ELS (serviceability limit states) or ELU (ultimate limit states);
- The locations or construction elements to be monitored (critical sections, deck, piles, bearings, abutments, etc.) and the stresses to which they are exposed;
- The environment and other variables affecting durability and integrity, as well as aging.

Consequently, this pilot has been designed as a flexible, adaptable, and scalable solution for remote BIM contextualization [38] of measurements with IoT systems, which is considered the most suitable and feasible technological approach for general or wide-use bridge monitoring in practice.

#### 3.1.2. Digital Components: On-Premises and Cloud System

On the on-premises side, the middleware ingests the raw sensor data stream via MQTT and performs real-time processing to detect vibration events caused by passing trains. When a threshold is exceeded, indicating a train event, the middleware isolates the free vibration segment of the signal and performs a fast Fourier transform (FFT) [39] analysis on it to identify the prominent vibrational frequencies. For this response (free vibration), the structure can be considered to be in a linear regime, as its natural frequencies only depend on the stiffness and mass matrix, but there is a transient character in the signal that has a short duration and requires a high sampling frequency (covered by the selected network and the transmission system) to be processed, and then we must deal with higher uncertainty in modal identification (covered by the proposed machine learning strategy). At the end of each day, the historical on-premises data are forwarded to the cloud data lake to augment the training dataset and continually improve the machine learning model’s accuracy. On the cloud side, the daily batch of FFT peak data from the bridge is stored in a data lake. Using Spark clusters, these data are transformed into consolidated, cleansed datasets suitable for machine learning. The key steps are:

- Filtering outliers in the vibration frequencies that fall outside expected ranges;
- Extracting the top three principal vibration peaks for each bridge crossing event;
- Clustering the data using k-means [25] to group similar vibration patterns;
- Labeling the clusters to create a supervised training dataset.

This curated dataset is used to train and re-train machine learning models to classify vibration patterns using automated machine learning and MLOps [40] techniques. The best-performing model is eventually deployed to an inference endpoint that can be called by the on-premises middleware. When the middleware sends new vibration peaks to the cloud endpoint, the model classifies the peaks into one of the learned clusters. This cluster assignment is returned to the middleware and stored. Any anomalies or changes in the typical cluster distribution can indicate a potential structural issue requiring further inspection. The on-premises database, in addition to the real-time inference results, registers contextual sensor data such as temperature.

### 3.2. Technical Details of Key Components

**Figure 2:**
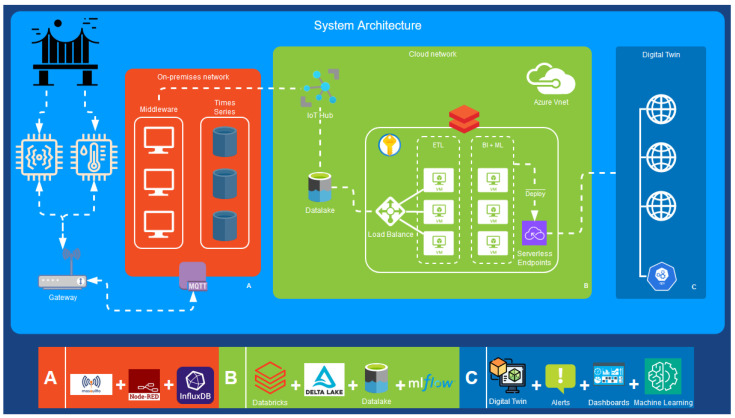
Components of the hybrid on-premises and cloud network architecture.

The structural health monitoring system follows a hybrid edge–cloud architecture to enable real-time monitoring and analytics. The key components include sensors and gateways as the edge layer, on-premises middleware for preprocessing, cloud services for storage and machine learning, and a digital twin application for data visualization and alerts. Figure 2 depicts the main components of the architecture. The components, as shown in the figure, are:

- Sensors: Wireless accelerometers installed on the bridge to measure vibrations. They stream data via WiFi to an on-site gateway.
- 4G gateway: Collects and transmits sensor data from the bridge location to the central system. Provides local WiFi connectivity.
- MQTT broker: Message queuing protocol used for efficient sensor data transmission.
- On-premises network (A): Middleware hosted on-site for real-time data ingestion and processing. Stores data in time-series database and runs analytics like FFT.
- Cloud network (B): Cloud services for scalable storage, batch processing, and machine learning. Batch data are ingested to the cloud storage data lake and processed in Databricks for ML model training. It includes MLflow, which enables machine learning model management workflows. MLflow is used to track, version, and deploy machine learning models into production in a serverless, scalable way.
- Digital twin application (C): Consumes real-time sensor data and includes a bridge geometry model for visualization and alerts. Includes dashboards, notifications, and a digital twin BIM viewer.

The edge layer acquires high-frequency sensor data, the on-premises middleware (A) handles real-time processing needs, while the cloud (B) provides big data and machine learning capabilities. The digital twin application (C) fuses sensor data with the bridge information model to bring monitoring insight to users.

#### 3.2.1. Mems Accelerometers

Low-cost SHM monitoring systems offer good behavior in terms of resolution, noise level, and sensitivity [41]. The MEMS [25] accelerometers used in the bridge monitoring system are the cost-effective BeanDevice Willow AX-3D [42] wireless vibration sensors. These triaxial accelerometers can measure vibration along three perpendicular axes, capturing the full motion of the sensor. The selected measurement range was ±2 g, meaning they can detect accelerations up to two times the force of gravity in any direction. The frequency response reaches 800 Hz: suitable for capturing bridge structural vibrations, which typically range from a few hertz to 50 Hz. The Willow sensor was configured to sample acceleration values at 500 Hz (a short signal of a few seconds needs a high sampling rate to have enough points to be processed by FFT) and streams the data over WiFi using a local network set up specifically for the bridge deployment. A 4G cellular modem, acting as the gateway, provides the WiFi network access to a wide area network (WAN). The accelerometer data are published to an MQTT message broker hosted on the 4G modem itself, minimizing external dependencies. Using MQTT allows the low-power wireless sensors to easily integrate with the rest of the monitoring system architecture.

#### 3.2.2. On-Premises System

The middleware was designed to handle the real-time processing and storage of sensor data. We chose the MQTT protocol for communication between the sensors and the middleware due to its lightweight nature, low bandwidth usage, and suitability for IoT and SHM applications [28].

For MQTT message brokering, the open-source Mosquitto [43] broker was utilized. Mosquitto is a scalable implementation of an MQTT server. The sensors were configured to publish their acceleration data streams to topics on the Mosquitto broker. The on-premises middleware was built using Node-RED [44], which is a visual programming tool for wiring together IoT devices, APIs, and services.

The use of Node-RED as middleware provides a cost-effective and flexible solution for industrial applications [45]. Custom Node-RED nodes were developed to decode the hexadecimal payload of the sensor messages into numerical acceleration values. Additional nodes enabled the storing of real-time data into an InfluxDB [46] time-series database. The Node-RED nodes enabled the following features for real-time analysis:

- Signal processing algorithms: Signal processing is a crucial aspect of our study, as it allows us to extract valuable insights from the raw sensor data collected from the railway bridge. In this study, we used a combination of filtering and noise reduction to process the high-frequency sensor data in real-time.
- Fast Fourier transform (FFT) analysis: Following filtering and noise reduction, the sensor data were then subjected to FFT analysis. FFT is a signal processing algorithm that transforms a signal from its original time domain to a representation in the frequency domain.

In our study, FFT analysis was performed on-premises using a custom-built Node-RED node. This tool was designed to handle the high data rates of the sensor data and performs FFT analysis in real-time, thereby enabling us to detect any changes in the bridge’s Eigenfrequencies after a train has passed. The Node-RED environment provided an agile way to stream and process the high-frequency sensor data on the middleware.

#### 3.2.3. Cloud System

The cloud infrastructure provider used was Microsoft Azure [47]. Within Azure, a Gen2 storage account was leveraged as a scalable data lake [48] for storing the historical vibration data forwarded from the on-premises system. Built on top of this data lake, a lake house [49] architecture was implemented using Databricks [50] for Azure. Databricks provided both data engineering capabilities for batch data processing as well as machine learning workflows in two workspaces.

In the data engineering workspace, Databricks was used to process the raw FFT data from the accelerometers. Spark clusters running on Databricks consolidated and cleaned these data, ultimately clustering the FFT spectra into three labeled groups representing different vibration patterns. For data transformation, the medallion architecture [51] was used. This transformed the raw data into curated silver and gold datasets to make them suitable for training machine learning models.

The machine learning component of Databricks leveraged the AutoML [52] feature to automatically train and evaluate different algorithms on the labeled FFT data. The best-performing algorithm was selected and registered into the MLflow [40] tracking server. MLflow enabled MLOps on Databricks by managing the model lifecycle, including model versioning, staging, and production deployment. Trained models were first tested in a staged environment and then promoted to production deployment on a serverless and scalable Azure Kubernetes service. The production models were exposed as real-time inference endpoints that could be called by the on-premises middleware system.

### 3.3. Implementation Challenges and Solutions

**Figure 3:**
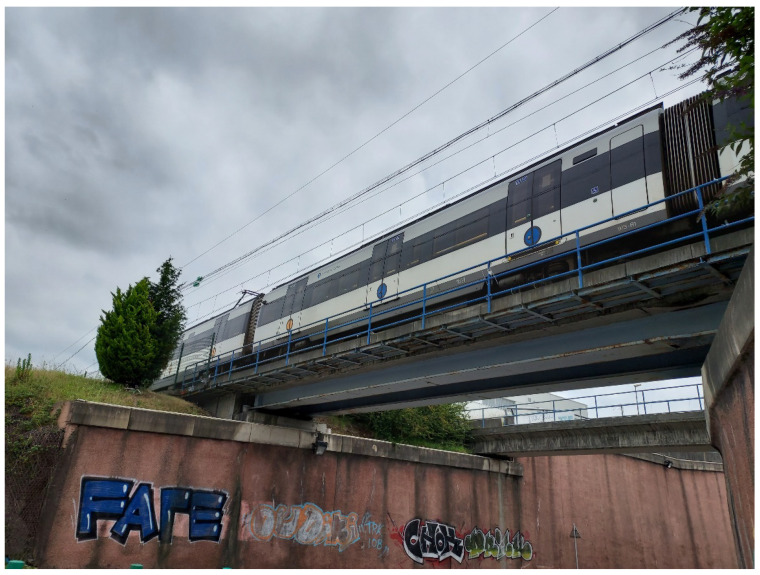
Railway bridge used as pilot at Torrelarragoiti industrial area, exploited by ETS Euskal Trenbide Sarea/Basque Railway Network (Zamudio, Basque Country, Spain).

With the support of ETS, Euskal Trenbide Sarea/Basque Railway Network through the company DAIR Ingenieros, the bridge selected for this pilot is located at PK11/520 on the Txorierri line in the industrial area of Torrelarragoiti (Zamudio, Basque Country, Spain), as shown in Figure 3 below. The characteristics of the structure under study are as follows:

- Railway bridge;
- A single isostatic span of approximately 17 m;
- Two non-standard steel main girders with reinforced concrete top slab.

The 17 m single-span bridge chosen as the pilot for this structural health monitoring system represents a typical medium-length span used for urban bridges. In this kind of single-span bridges, deterioration issues eventually emerge from accumulated traffic, material aging, and exposure to climatic conditions.

In order to record as clearly and accurately as possible the first three vibration frequencies, an accelerometer was placed close to the span center (to contrast the first longitudinal bending mode and, as a local variable, the acceleration of the point of maximum displacement, since this acceleration can also be controlled by a direct threshold), an accelerometer was placed at one-fourth of the span (between the span center and support, approximately where the maximum amplitude of the third modal form of vibration is located, corresponding to a second longitudinal bending, to which the accelerometer at the span center is theoretically blind), and an accelerometer was placed on the other beam to characterize the torsion (second theoretical mode, which might not always be excited by the passing of the centered load). However, this last added sensor ended up being removed before the end of the installation, as the concrete slab shielded the coverage provided by the WiFi antenna on the other side of the beam. That is, only one of the main girders was instrumented, which was sufficient for the demonstration or functionality test of the system parts once assembled, which is the objective of the pilot test. The installation was completed in about 3 h, but it may be carried out faster on more accessible bridges. It was necessary to make a cut in the road under the bridge and use a flatbed truck to access the flange of the main girders. It was decided to place the gateway and the accumulators on the flange and to place the sensors next to the web. According to the usual practice of instrumentation of structures, this is a suitable position for the measurement of accelerations and main vibration frequencies.

It is important to emphasize that it is necessary to record measurements from bridges for further analysis (a complete dynamic and/or more detailed study can be done by an expert after downloading data from the platform) and to have a power outlet available. Failing that, it is necessary to install solar panels and their respective accumulators, since the measurement equipment generally cannot withstand many hours without power when high measurement and transmission rates are required. IoT equipment that has autonomy for long periods of time using only batteries usually measures and transmits data every several minutes, while for the pilot experience, up to 500 data points per second were being recorded for train passages (achieved by implementing communication through the MQTT protocol and a 4G network).

Regarding data quality, the span of the bridge resulted in being a bit short, and the free vibration of the bridge was restricted to less than 8 s. This meant that the part of the signal (after the train exit) corresponding to the free vibration had to be precisely captured. To solve this, and since the train units always cross at the same speed, a manual time adjustment of the capture was configured in the middleware to ensure that a significant free vibration signal was captured for each crossing.

Another issue came from the differences in temperatures from one day to another. The sensors were sensitive to the temperature and summed an offset in the signal, generating false triggering due to detection of signals that exceeded the RMS thresholds and activating the capture of invalid signals. In addition, under certain climate circumstances, some noise was induced due to interference that occasioned the same triggering issue. The solution comprised regular remote calibration of the MQTT sensors when notable temperature changes were forecast.

Concerning the selection of the on-premises and cloud infrastructures, a key challenge was to find the right balance for leveraging both platforms cost-effectively. While cloud services excel at data storage, batch processing, and machine learning, they can become prohibitively expensive. For this bridge monitoring use case, the raw sensor data rates were too high to process solely in the cloud. By handling real-time ingestion, parsing, and FFT analysis on-premises, the data forwarded to the cloud were reduced by orders of magnitude. This avoided excessive cloud data ingress charges. However, the cloud was still leveraged for its strengths, such as cheap storage, distributed batch data processing, and on-demand machine learning model training and deployment. These tasks are challenging to implement on-premises due to the maintenance costs and configuration efforts of these components. To scale the solution to many bridges, the on-premises components allow cheap real-time analytics, and the cloud then enables aggregated analytics by consolidating data from all bridge deployments. Careful testing was conducted to find the optimal split between real-time processing and batch cloud analytics. This balance minimized costs while still providing a robust, low-latency structural health monitoring system.

## 4. Experimental Methodology

### 4.1. Generic Methodology

This sub-chapter introduces a comprehensive, generic methodology for structural health monitoring (SHM) of railway bridges that leverages low-cost IoT sensors, hybrid edge–cloud processing, finite element model (FEM) calibration, unsupervised learning to identify normal vibration patterns, generation of synthetic damage data, and supervised model training and validation. The goal is to enable accurate, cost-effective, and scalable SHM that can be replicated for other pilot bridges beyond the specific one presented in this manuscript. The proposed ideal framework consists of seven key steps:

1. Characterization of the railway bridge;
2. Sensor deployment and data acquisition;
3. Data preprocessing;
4. Unsupervised learning;
5. Model calibration and synthetic data generation;
6. Supervised learning;
7. Validation and continuous learning.

While not all seven steps were fully implemented for the pilot bridge in this work, the overall methodology provides a complete reference that other researchers can adapt and build upon to achieve robust SHM using affordable sensing and advanced analytics. The specific application and customization of this generic approach for the pilot railway bridge is detailed subsequently in Section 4.2.

By presenting this methodology, the aim is to establish a replicable foundation for data-driven SHM that fuses IoT, AI, and digital twins to deliver a proactive, automated solution for assessing bridge health at a fraction of the cost of traditional inspection-based or wired monitoring techniques. This will allow infrastructure managers to scale real-time damage detection across their entire bridge networks: enabling timely maintenance interventions that prevent catastrophic failures and extend structure lifespans.

#### 4.1.1. Characterization of the Railway Bridge

The proposed generic methodology begins with thoroughly characterizing the railway bridge through finite element method (FEM) modeling and detailed in situ measurements. A parameterized 3D FE model of the bridge should be created in software like ANSYS using the parametric design language (APDL). This virtual FE model should capture the precise geometry, structural elements, material properties, and existing damage conditions of the physical bridge. To achieve this, the in situ measurements should involve meticulously recording the bridge dimensions, cross-sections, material types (steel, concrete, etc.) and visible degradation.

Although it has not been carried out in the present work, in order to achieve a faithful characterization of the structure, ideally, the measurements may involve testing to obtain concrete strength, rebar details, etc. These measurements ensure the FE model accurately represents the real bridge.

As a result, at least the first three principal vibration mode shapes and frequencies should be calculated from this initial FEM bridge model. These will serve as a baseline for subsequent measurements and model calibration.

#### 4.1.2. Sensor Deployment and Data Acquisition

Low-cost MEMS wireless accelerometer sensors should be configured for optimal sampling rates (e.g., 500 Hz) based on the expected vibration ranges and response duration from train loading. The sensors should be ruggedized with IP67 rating for outdoor conditions and integrated with solar panels and rechargeable batteries for self-powered operation.

In our work, we implemented an optimized sensor installation plan by placing the accelerometers at the best locations on the bridge based on the FE analysis. The sensors connect to a local 4G gateway on the bridge through a WiFi mesh network, enabling redundant communication paths. The gateway streams the high-frequency vibration data to an on-premises middleware system using the MQTT protocol over 4G/5G networks. Triggers were set to activate data capture only during train crossing events in order to conserve bandwidth and battery life.

#### 4.1.3. Data Preprocessing

The raw vibration data should be preprocessed in the on-premises middleware by applying filtering and outlier removal techniques to improve signal quality. After each train crossing, the free vibration portion of the signal should be extracted; this corresponds to the linear regime when only the bridge’s natural frequencies are present. Fast Fourier transform (FFT) analysis should be applied to this free vibration data to identify the prominent vibration frequency peaks, with a focus on the three principal peaks that correspond to the fundamental mode shapes.

#### 4.1.4. Unsupervised Learning

The empirical vibration data, consisting of the three principal frequency peaks from each train crossing event, should be clustered into distinct groups using the k-means algorithm. This unsupervised clustering method segments the data into normal operating condition clusters based solely on the vibration patterns. To confirm cluster stability, it must be ensured that the relative sizes of the clusters do not change significantly as more data are accumulated over time. This would confirm the system is able to learn the intrinsic vibration patterns. Next, the data points should be labeled based on their assigned cluster for subsequent supervised learning.

#### 4.1.5. Model Calibration and Synthetic Data Generation

The FE bridge model should be calibrated by tuning parameters like the modulus of elasticity of the steel or the concrete, or the boundary conditions (not always ideal), to match the empirical vibration modes extracted from the sensor data under normal operating conditions. Thus, this calibrated FE model would act as an accurate digital representation of the real bridge. Synthetic damage scenarios should be simulated in the FE model by reducing parameters like the elastic modulus of steel or the inertia of components by a small amount, like 10%, 20%, or 30%, based on, e.g., visual degradation observed during inspections. Finally, new vibration modes should be calculated from this analytical damaged state.

The percentage differences between the damaged and normal vibration modes should be applied to the empirical sensor data clusters to create the same number of corresponding synthetically damaged data clusters. These should be labeled differently from the normal condition clusters.

#### 4.1.6. Supervised Learning

The normal and synthetic damaged labeled datasets should be combined into one dataset with the total number of clusters: the normal operation clusters and the corresponding damaged data clusters. This consolidated dataset should be split into train, test, and validation partitions. Several machine learning classifiers, like the LightGBM classifier finally selected for this bridge, should be trained on the train set using the AutoML approach to automatically tune hyperparameters like tree depth, learning rates, etc., via cross-validation on a validation set. Metrics like log loss, receiver operating characteristic curve and area under the curve (ROC-AUC), precision, and recall should be systematically tracked to optimize the model’s discrimination ability.

Once the optimal model configuration is identified, it should be evaluated on the held-out validation set to estimate the final classification accuracy, precision, recall, and F1-score for detecting abnormal/damaged vibration patterns against normal patterns using the empirical sensor data.

#### 4.1.7. Validation and Continuous Learning

The final stage of our generic methodology, validation and continuous learning, is crucial for maintaining the integrity and relevance of our structural health monitoring system. The validation process, therefore, confirms not only the model’s accuracy but also its reliability in a real-world setting.

After training the machine learning model on the labeled dataset of normal and synthetic damaged vibration patterns, it is crucial to rigorously validate the model’s performance and accuracy. This validation step assesses the model’s ability to generalize and correctly classify new, unseen vibration data from the bridge sensors into the appropriate normal or anomalous clusters. Once validated on this test set, the trained model should be deployed to a production environment for real-time inference.

Our trained LightGBM model achieved an excellent validation ROC-AUC score of 0.9999, demonstrating its ability to accurately discriminate between normal and anomalous vibration patterns on the validation set. In the production system, this model is deployed to a serverless, cloud-based inference endpoint. The on-premises middleware streams new vibration data from the bridge sensors to this endpoint for real-time classification. The model’s predictions along with sensor metadata like temperatures are stored in the on-premises database. Any anomalies or deviations from the typical cluster distributions can provide early warning of potential structural issues requiring further inspections.

However, the monitoring process does not stop there. This validated model now becomes the initial baseline that enables continuous learning and adaptation as more real-world vibration data accumulate over time. The digital twin system stores all historical sensor data in a cloud data lake. At regular intervals, this growing dataset is used to retrain and update the machine learning model, further enhancing its accuracy through additional training on real-world examples. This continuous learning loop allows the model to automatically adapt to gradual changes in structural dynamics, seasonality effects, aging of the bridge materials, and more.

### 4.2. Application of the Methodology to the Pilot Bridge

#### 4.2.1. Overview of Field Deployment

The structural health monitoring system was deployed on an in-service railway bridge for an extended real-world validation. The instrumentation was installed rapidly, as the equipment consists mainly of wireless devices. Despite exposure to weather and vibrations, the ruggedized equipment required no maintenance over two years of continuous operation. Over this period, the system captured a rich dataset of bridge vibrations under daily rail traffic with a mean frequency of two trains crossing every 18 min. The machine learning models were successfully trained on these data to classify normal vibration patterns, and the model deployment demonstrated the effectiveness and robustness of the monitoring system concept. The sensors provided reliable high-frequency data even in noisy environments near active rail lines.

The digital twin concept demonstrated here proved to be robust, and the implementation methodology can be extended to other railway bridges with similar characteristics and potentially to other types of infrastructure.

#### 4.2.2. Sample Acceleration Data Collected from Sensors

There is some variability in the natural frequencies extracted during each train pass because different trains pass in both directions with slightly varying loads and speeds that do not excite the structure in the same way, and also, there is inherent variability due to working with short free vibration signals rather than long environmental vibration recordings. To cope with this fact, a clustering algorithm was used to separate the measurements into a reduced number of groups or cases. Basically, depending on the passing of the train, some frequencies are not identifiable after the departure of the vehicle because they have not been sufficiently excited, or signals with higher noise and/or insufficient duration of free vibration are generated for a correct processing. These latter signals are discarded, since the identification of frequencies with accelerations of free vibration is more unstable with these more limited sensitivity sensors (lower than class A ones). That is, a series of clusters can be separated to generate three different clusters. With clustering, different regions of normality can be easily defined for the training of anomaly detection so that once the algorithm has been trained, at the passage of each new train, the membership of each of the clusters can be evaluated.

**Figure 4:**
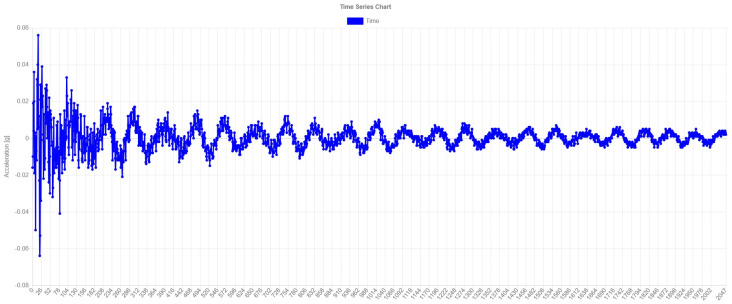
Time-series plot of the free vibration after the crossing of a train.

Figure 4 shows a time-series plot of free vibration after a passing train. The signal is further processed with an FFT algorithm for frequency domain transformation and classification.

**Figure 5:**
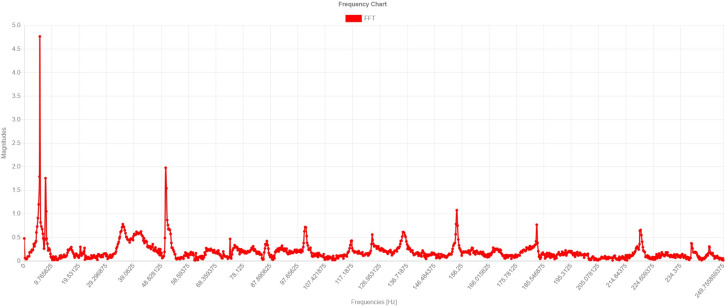
Frequency plot of the free vibration after the crossing of a train.

Figure 5 shows a plot of the free vibration FFT of the previous figure. Clustering is performed based on the patterns of these modal frequencies for the characterization of the normal situation of the bridge.

To validate this characterization, the free vibrations were processed after the passing of a train to obtain a vector with the natural frequencies of the structure of the pilot research case. These frequencies are approximately in agreement with the frequencies of the Ansys Mechanical APDL [53] finite element model, knowing that this is only indicative because there are no drawings of the structure and was no access permission to carry out exhaustive measurements. Thus, it was verified with regard to the frequency domain that the measurements provided by the equipment are valid and that the bridge does not differ appreciably from its theoretical behavior, i.e., as it is physically.

After assigning some parameters that could not be measured directly within acceptable values (Young’s modulus of steel = 210,000 MPa, Young’s modulus of concrete = 35,000 MPa, density of steel = 7850 Kg/m^3^, density of concrete = 2400 Kg/m^3^, and web thickness of the non-standard steel girder = 65% of the measured flange), the values obtained by the finite element model for the main three vibration modes are as follows:

- 5.71 Hz for the first mode (bending);
- 15.11 Hz for the second mode (torsion);
- 20.84 Hz for the third mode (second order bending).

**Figure 6:**
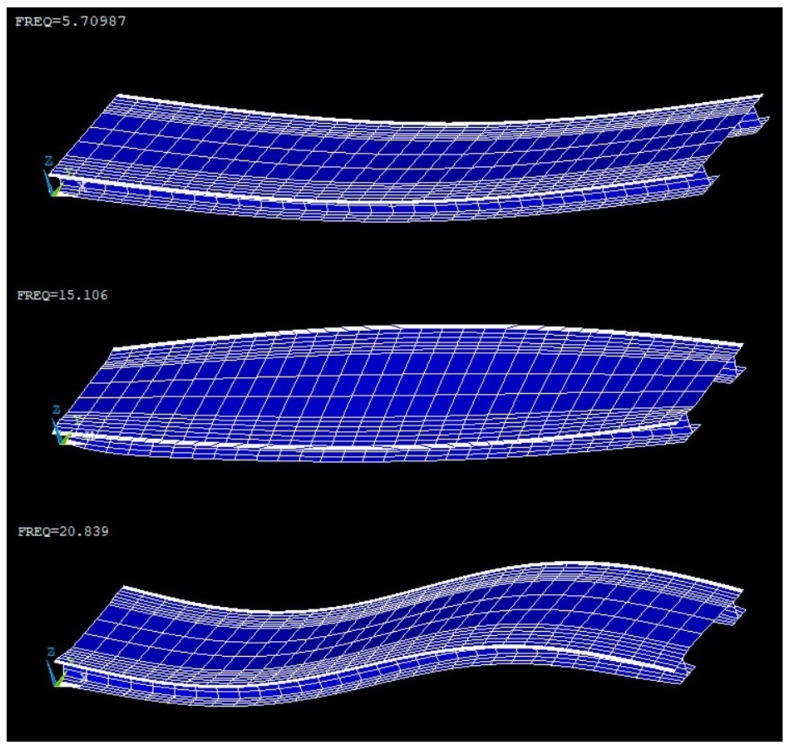
Modal shapes of the Ansys Mechanical APDL for the pilot bridge. The boundary conditions are those of a simply supported beam (applying the restraints at the bottom flange end of the steel girders in line with how the structure is actually supported).

The corresponding modal shapes are shown in Figure 6 (Ansys Mechanical APDL).

### 4.3. How a Digital Twin Detects Structural Changes from Sensor Data

Over the 2 years of testing, the system collected around 4300 valid samples of bridge vibration spectra during train crossing events. Table 1 shows a characterization of the collected FFT data.

**Figure 7:**
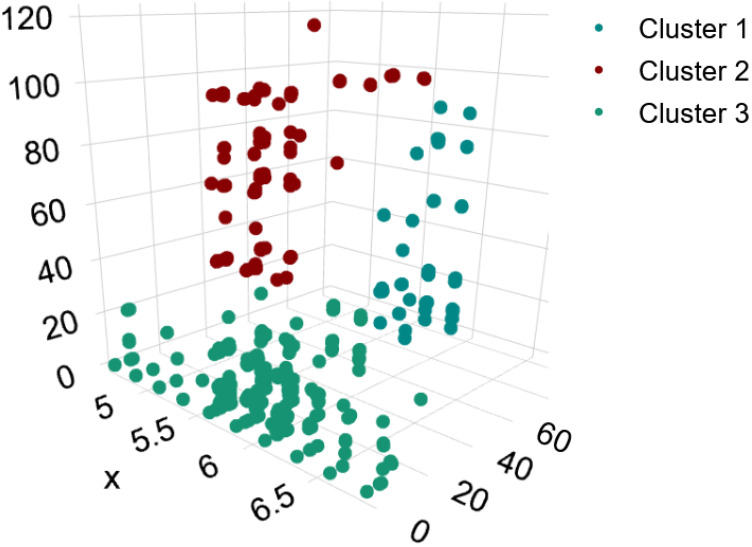
Distribution of the three clusters in the 3D space.

Using k-means clustering on this FFT data, the digital twin consistently identified three distinct clusters representing normal vibration patterns. Figure 7 illustrates the distribution of the three clusters in a 3D space, along with a descriptive analysis of the data. The relative sizes of the three clusters did not change as more data were accumulated over time, confirming that the system learned the three intrinsic vibration patterns characterized by the clusters.

Table 2 and Table 3 show the distribution of the cases into clusters, the description of the cluster center positions, and the statistical characterization of the three clusters.

**Figure 8:**
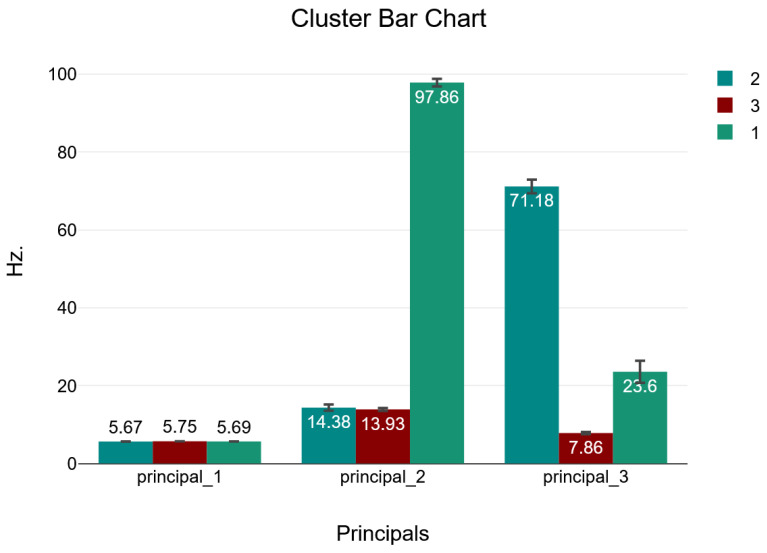
Cluster mean values and standard deviations.

Figure 8 summarizes the distribution of the total samples across these three clusters identified under normal operating conditions, providing insight into the prevalence of different vibration signatures in the raw acceleration data.

According to the provided data, the 4323 cases were distributed as follows:

- Cluster 1 contained the fewest cases at 190 cases.
- Cluster 2 had 659 cases.
- Cluster 3 was the largest group with 3474 cases.

The dominance of Cluster 3 indicates that nearly 80% of the observed vibration patterns fell into this category. Cluster 2 had the next highest portion at 15% of the cases, while Cluster 1 represented about 5% of the measured spectra. This distribution shows that most of the vibration data (95%) were classified into Clusters 2 and 3, which likely represent the fundamental natural frequency of the bridge and low-order resonances. The fewer members of Cluster 1 suggest it corresponds to less common transient or higher-order vibration phenomena.

The clusters represent distinct vibration frequency patterns observed during train crossing events. Clusters 3 and 2, with a peak frequency around 5 Hz, likely correspond to the fundamental bending mode of the bridge span, and the peak frequency of 14 Hz corresponds to the torsion mode. Cluster 1 probably captures higher-order resonances such as high-order bending modes, including transient vibrations without clear modal patterns. By separating the raw acceleration signals into these characteristic clusters, the digital twin model establishes baseline behavior profiles for the bridge.

The relative size of the clusters quantifies the prevalence of different vibration signatures identified by the machine learning model under normal operating conditions. Tracking changes in the cluster distributions over time then enables assessing shifts in the structural response that may require further inspection. This cluster analysis demonstrates the digital twin can characterize the normal response in terms of Eigenfrequencies from the raw acceleration signals.

Under the assumption used in other works [32], it is possible to generate damage in the computational model and consider the variation ratio it causes in the vibrational parameters. Calculating the ratio—the relative change between the initial (healthy) and the damaged scenario—eliminates the effect of modeling error and uncertainty for validation. That is, the calculated variation ratio is subsequently applied to the real signals, assuming that it is constant over the short period of time considered for testing. With this approach, we built realistic damaged data that include the variability of experimentally measured signals and use them to validate the performance of the anomaly detection algorithm.

Thus, given that there is no precise information and that the deterioration of the bridge could be associated more with its general aging, a synthetic damage scenario was generated with a 10% loss in the modulus of elasticity of the girder’s steel, thus obtaining an average variation ratio (decrease) of the Eigenfrequencies of around 3%. This is not intended to be a recreation of a real damage scenario but, rather, provides an order of magnitude of variations to test the method.

As a consequence, new vibration modes were calculated from this simulated FEM state by applying the variation ratio of 3% between the damaged and normal vibration modes to the empirical sensor data clusters to create three corresponding synthetic damaged data clusters. The normal and synthetic damaged labeled datasets were combined into one dataset with a total of 8646 rows of data associated with the six clusters: three normal operation clusters and three corresponding damaged data clusters. The clusters were labeled as: ok_cluster_1, ok_cluster_2, ok_cluster_3, damaged_cluster_1, damaged_cluster_2, and damaged_cluster_3. This consolidated 8646 row dataset was split into 60/20/20 train/test/validation partitions.

Leveraging MLflow capabilities, the cloud machine learning model was trained using AutoML to classify any new vibration data into one of these six clusters. Databricks AutoML was leveraged to automatically evaluate different machine learning algorithms and hyperparameters. The classification models tested included decision trees, random forests, logistic regression, XGBoost, and LightGBM. An MLflow experiment was launched that ran various combinations of these algorithms with tuned hyperparameters. At the conclusion of the experiment, the best-performing model was selected based on accuracy metrics. The hyperparameters of the LightGBM model selected were:

- colsample_bytree = 0.6317331055500884;
- lambda_l1 = 0.1459367945385852;
- lambda_l2 = 0.30720685012169846;
- learning_rate = 0.03691431372131188;
- max_bin = 264;
- max_depth = 5;
- min_child_samples = 36;
- n_estimators = 551;
- num_leaves = 7;
- path_smooth = 95.01320854755053;
- subsample = 0.5638297006431046;
- random_state = 190,645,121.

The optimal model proved to be a LightGBM classifier automatically trained using the scikit-learn Python library. LightGBM can rapidly train classification models, and with its blend of performance, accuracy and scalability, it has become a popular machine learning package suitable for a wide range of predictive modeling applications from analytics competitions to production systems [54]. The structural health monitoring system utilizes this classifier due to its high performance in training models on large vibration datasets to accurately classify normal versus abnormal bridge conditions. The evaluation metrics for the chosen LightGBM model were:

- Training data and splitting:
  - The model was trained on 8646 rows of data;
  - A 60/20/20 train/validation/test split was used;
  - This means 5188 rows for training and 1729 rows each for validation and test sets.

- Best iteration (281) and stopped iteration (286):
  - The model achieved its best validation performance after 281 boosting rounds;
  - Training was stopped early at iteration 286, which was close to the best iteration;
  - This indicates the early stopping callback worked well to prevent overfitting.

- Test metrics:
  - Test log loss: 0.0120—very low, indicates high accuracy on unseen test data;
  - Test ROC-AUC: 0.9999—near perfect discrimination ability on the test set.

- Training metrics:
  - Training log loss: 0.0099—extremely low, model fits training data remarkably well;
  - Training ROC-AUC: 0.9999—near perfect discrimination on training data;
  - Potential for some overfitting, but this is not concerning given test performance.

- Validation metrics:
  - Validation log loss: 0.0171—low, but higher than training, as expected;
  - Validation ROC-AUC: 0.9999—excellent discrimination on validation data;
  - Valid_0 log loss: 0.0171—same as overall validation for the first fold.

In summary, the model achieves very high accuracy, with near perfect discrimination as measured by the ROC-AUC on both the validation and held-out test sets. The early stopping callback effectively prevented overfitting. The low log loss values indicate the model is well calibrated for its predicted probabilities. The consistency between validation and test metrics is a good sign that the model generalizes well to new data.

By leveraging AutoML to automatically find the best classification algorithm through hyperparameter tuning, we were able to build an accurate machine learning model for detecting anomalies in the vibration data using the labeled training clusters. The model serves as the predictive engine behind the digital twin’s structural health analysis capabilities. By continuously analyzing the stream of new accelerometer data, the model could detect when the vibration patterns deviated from the normal clusters, i.e., shifting from ok_cluster_1 to damaged_cluster_1 with a probability >99.9%, accounting for a detection resolution capable of detecting a 10% synthetic reduction in the steel’s elastic modulus.

To quantify the extent of the deviation, we defined a “damage index”, which is easily interpretable by the end user, as the probability that a vibration data sample does not belong to any of the three known normal clusters and belongs to the corresponding damaged cluster. The higher this index, the more dissimilar the vibrations were from patterns seen during normal operation.

**Figure 9:**
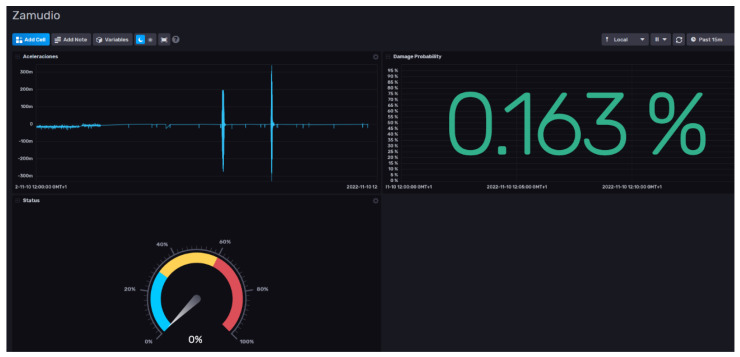
Dashboard implementation with “damage index” or probability of failure.

Figure 9 shows a dashboard implementation with the “damage index” or probability of failure after train passing. The reliability of this result increases with the number of data points collected and the number of variables included.

These clusters established a profile of expected normal structural dynamics. By continuously analyzing new data, any vibrations that deviated from the learned clusters could indicate a change in the bridge’s condition.

For example, changes in vibration magnitudes (for a fixed input, i.e., the train) detected in certain frequency bands could suggest a loss of structural stiffness, as would a reduction in the main Eigenfrequency (abscissae of the FFT peaks) values. Also, new vibration peaks appearing could imply damage to the structure, like the prolongation of the vibration after a train crossing (due to changes in structural damping).

While controlled damage testing was not possible on the active railway bridge, the long-term monitoring data itself revealed seasonal shifts in vibration patterns due to factors like temperature. The digital twin was able to adapt and maintain high accuracy despite these operational variations. To cope with these seasonal shifts, the middleware and cloud platform implemented a mechanism that filtered out the outliers in the vibration registries that fell well outside the expected ranges. The correction of this issue was carried out manually by accessing the sensors’ remote interfaces and adjusting the sensors’ offset.

**Figure 10:**
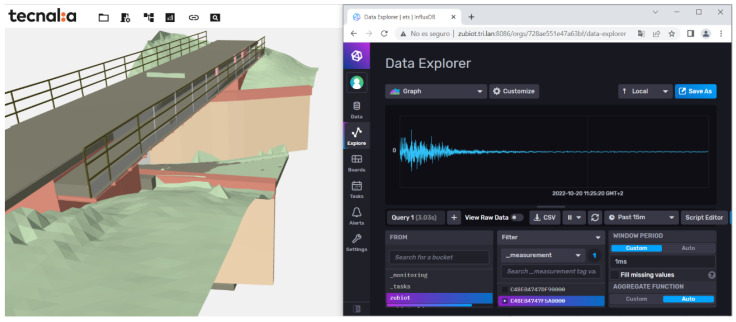
Interactive digital twin of the bridge from a BIM model (via IFC file) including real and historical sensor data on top of user-selected elements.

**Figure 11:**
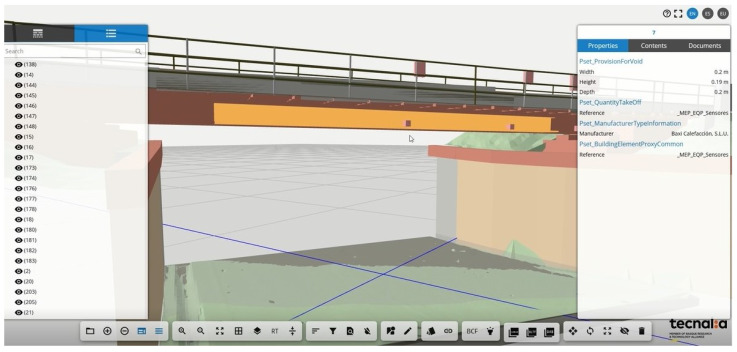
Interactive digital twin of the bridge from a BIM model (via IFC file) including an index to access the properties of user-selected elements.

Regarding user interaction and visualization, a BIM model of the bridge, generated by RDT Ingenieros, and the sensors was built. The model enables a common data environment wherein the geometric, structural, and sensor data are accessible and linked together as a digital twin. Included in the features of the visualization system is a train crossing visualization, as shown in Figure 10 and Figure 11, for which the free vibration of a crossing is shown in real-time.

Another set of functionalities includes a system for sending alerts based on thresholds configured by expert users and a dashboard with the probability of failure after a train passing. In essence, the digital twin learned an allowable range of normal vibrations over long-term monitoring through machine learning. This enables it to detect abnormal patterns that are potentially linked to structural changes. The system provides a data-driven method for continuously assessing bridge health without requiring baseline physics models.

## 5. Results and Discussion

### 5.1. Summary of Digital Twin Approach for SHM in Railway Bridges

This pilot study demonstrated the capabilities of a digital twin solution for real-time structural health monitoring of railway bridges using low-cost wireless accelerometers. The system leverages a hybrid edge–cloud architecture to efficiently process and analyze vibration data for anomaly detection, indicating potential structural issues or damage.

The wireless MEMS accelerometers, costing only EUR 30–300 per unit, enabled a significant cost reduction compared to traditional wired sensors, which often cost more than EUR 3000 each. This cost effectiveness allows for large-scale deployments across thousands of bridges at a fraction of the cost of conventional wired systems. The self-contained design with integrated battery or solar power sources eliminates the need for the extensive cabling and power infrastructure associated with typical SHM deployments. This simplified installation process reduces complexity compared to conventional systems. Assuming that for a typical wired installation it would have taken approximately 16 h to prepare the wiring and perform an equivalent installation with standard sensors, including the connection and proper protection of the acquisition module and field PC (all this assuming that a power outlet was available, which was not the case), the proposed installation was completed in approximately 3 h, resulting in well over a 50% reduction in installation complexity.

Moreover, the maintenance burden is alleviated through the system’s remote management capabilities, which are facilitated by a digital twin interface. Sensor parameters and configurations can be adjusted remotely, eliminating the need for manual intervention at each device location. Additionally, the over-the-air (OTA) update functionality ensures sensors remain up-to-date with the latest firmware without requiring physical access. Assuming a typical SHM system requires 20 person-hours per year for maintenance, the proposed system is estimated to require only 5 person-hours per year, resulting in a 75% reduction in maintenance complexity.

The edge middleware handles high-speed data ingestion, preprocessing, and real-time FFT analysis, while the cloud provides scalable storage, batch processing, and machine learning capabilities. By performing computations at the edge and leveraging serverless cloud architectures, the system achieves substantial cost savings compared to traditional cloud-based solutions. For example, assuming a deployment of 1000 bridges with 10 sensors each, storing raw data in the cloud would cost $6900 per month, while storing processed data (10% of the size of the raw data) would cost only $690 per month, resulting in a 90% reduction in cloud storage costs.

The digital twin’s machine learning algorithms, which are trained on historical vibration data, achieved a classification accuracy of ≥99.9% for detecting the abnormal vibration patterns indicative of structural issues. The LightGBM model, optimized using AutoML techniques, demonstrated excellent performance in distinguishing between normal and anomalous vibration signatures. The model’s high accuracy enables early detection of potential damage before it becomes visually apparent during inspection.

Compared to state-of-the-art methods, the proposed digital twin solution offers several key advantages:

1. Cost-effectiveness: achieves lower costs than traditional wired sensor networks through the use of low-cost wireless sensors and edge–cloud computing.
2. Scalability: enables large-scale monitoring across thousands of bridges with minimal additional infrastructure costs.
3. Ease of deployment: reduces installation and maintenance complexity through the use of self-contained, battery/solar-powered wireless sensors.
4. High accuracy: attains outstanding classification accuracy for detecting abnormal vibration patterns using rapid advanced machine learning techniques.
5. Actionable insights: provides continuous, automated structural health assessment and generates maintenance recommendations to prevent failures.

### 5.2. Comparison to the Existing Literature

Comparing our results with the existing literature and systems using the same metrics, the article [55] demonstrates the use of support vector machine (SVM)-based algorithms for structural health monitoring (SHM) and achieves high classification accuracy. The focus of the study on SVM-based algorithms and their performance for classifying structural health states underlines the potential of machine learning techniques to enhance the accuracy of SHM systems. The article shows the results of a comparison of a basic SVM and enhanced SVM-MD, SVM-S2, SVM-SP, and SVM-EN (SVM based on ensemble classifiers) for simple and reinforced concrete (RC) beams. The results evidence a significant difference in performance between the basic and the enhanced SVMs, with SVM-MD outperforming the basic SVM with an accuracy of 86.29% for RC beams. Furthermore, SVM-EN achieved the highest accuracy among the proposed algorithms, with 87.2% for simple beams, demonstrating the potential of ensemble learning for improving classification performance.

The article [56] further supports the feasibility of achieving high classification accuracy for detecting abnormal patterns using machine learning techniques. The study found that the SVM algorithm was up to 94% accurate and precise for classifying data for historic building monitoring.

In terms of installation costs and maintenance complexity, the article [57] highlights that the low installation and maintenance costs of wireless sensor networks have made them a powerful alternative to traditional SHM systems that employ wired sensor networks. The referenced article presents comparative metrics, including deployment time and cost. Ref. [58] shows a real-world example wherein the deployment of a wired sensor network took several days, while the deployment of a wireless sensor network for the same example took only half an hour. Regarding costs, Ref. [59] demonstrates that a real-world implementation of a wired sensor network costs USD 10,000 to 25,000, compared to USD 500 for a wireless sensor network node. This supports the feasibility of reducing installation costs and maintenance complexity. The article [55] further contributes to this discussion by showcasing the effectiveness of machine learning models that can process data from wireless sensors, such as the transducers used in their experiments. The use of these wireless sensors simplifies the installation process and reduces complexity compared to traditional wired systems.

Moreover, the article [60] provides strong evidence that a well-designed and optimized hybrid edge–cloud architecture can lead to substantial cost efficiency improvements for IoT applications compared to using only cloud or only edge computing. The key enablers are local data processing at the edge, intelligent workload distribution, dynamic resource allocation, and cost-aware optimization algorithms. The evaluation results show that the proposed edge–cloud approach achieves significant cost savings compared to cloud-only or edge-only deployments.

The findings from the referenced articles support the target objectives and statements of our article. The high classification accuracy achieved by the enhanced SVM algorithms, particularly SVM-EN with 87.2% accuracy for simple beams, demonstrates the potential of machine learning techniques for detecting abnormal vibration patterns using low-cost wireless sensors. The cost and complexity reductions associated with wireless sensor networks and the benefits of a hybrid edge–cloud architecture further validate the feasibility of enabling scalable large-scale monitoring across thousands of bridges while reducing installation and maintenance complexity. By quantifying these improvements and comparing our results to the existing literature and systems, we can effectively demonstrate the advancements and benefits of our proposed digital twin approach for SHM of railway bridges.

### 5.3. Benefits Demonstrated: Low Cost and Rapid Damage Detection

The most significant achievements of the pilot application can be summarized as follows:

1. Establishment of a replicable methodology, with slight adaptations to each case study, for the generalization of railway bridge monitoring and its integration into the Internet of Things (IoT): The approach shown can be extended to the numerous similar bridges found in any metropolitan area network. As a result, the ability to monitor such common bridges is crucial for effective management of urban infrastructure health.
2. Identification and enabling of reliable MQTT communication with sufficient time density of measurements that allows for subsequent structural analysis of bridges (sent by a 4G network through an MQTT broker): This is also applicable to different sensing equipment—it has been possible with this communication protocol to integrate both low-cost and solar-powered commercial wireless systems (selecting the most competitive in price for the pilot) and standard universal acquisition modules for wired sensors. That is, the sensors to be used in the system can be selected according to the technical needs and the budget available for the monitoring of each bridge. With this combination of industrial-grade sensors, localized connectivity, and a standardized MQTT data interface, the solution can reliably collect high-frequency vibration data on the structural dynamics of railway bridges. The cellular modem connectivity enables remote data access from anywhere with internet access.
3. Development of a hybrid on-premises processing system using open-source tools that enables communication between hardware or physical devices and the cloud, allowing a high sampling frequency for each installed sensor: This system or data platform is extensible and scalable to any bridge with the corresponding slight adaptations to accommodate new sensor technology. The combination of Mosquitto, Node-RED, and InfluxDB 2.0 enables real-time collection, analysis, and storage of the acceleration data on-premises. Mosquitto handles collecting and distributing the massive amount of sensor messages. Node-RED processes and analyzed the data streams, and InfluxDB 2.0 acts as the time-series database for operational monitoring and data historians. On the cloud side, Azure Databricks provides a cloud platform for scalable data engineering, machine learning model training with AutoML, and robust MLOps for managing and deploying the models using MLflow. The cloud services provided the bandwidth needed for big data workflows on the historical vibration datasets. While this implementation used a LightGBM classifier selected by the Databricks AutoML functionality for vibration pattern analysis, the flexible architecture can adapt a wide range of machine learning and deep learning algorithms, as reviewed in the literature and including CNNs, RNNs, autoencoders, and other neural network architectures, into the SHM data pipeline as needed based on modifying the model’s requirement.
4. Ability to continuously learn and adapt over time as more real-world data accumulates: The validated AutoML machine learning model serves as an initial baseline, but it does not remain static. By storing all historical vibration data from the bridge sensors in a cloud data lake, the system gains access to an ever-growing dataset. At regular intervals, this expanding dataset is leveraged to retrain and update the machine learning model, steadily enhancing its accuracy through additional training on real examples from the field. This continuous learning capability allows the model to automatically adapt to gradual evolutions in the bridge’s structural dynamics, such as effects from seasonality, aging of materials, and other factors. Thus, the digital twin does not remain frozen but instead dynamically evolves its understanding of normal versus abnormal vibration patterns as conditions change over the bridge’s lifetime.
5. Visualization centered on a realistic digital twin with location and access to the information of each sensor that is represented as the physical reality and has assigned IDs: That is, IoT is integrated as a digital twin connecting streaming data, AI generated alerts, and the physical geometry of the bridge.

The hybrid architecture allows real-time edge analytics combined with cloud machine learning over historical data for an efficient, scalable structural health monitoring system. The middleware handles real-time needs, while the cloud provides flexible big data and machine learning capabilities. This architecture proved highly scalable, as additional bridges can be onboarded by deploying standalone on-premise systems for each. The centralized cloud platform aggregates and analyzes data from all bridges as needed.

The hybrid on-premises–cloud architecture was key to overcoming the implementation challenge of applying cost effective and replicable machine-learning-based monitoring to high-frequency sensor data from the infrastructure.

The successful pilot proves the field viability and paves the way for expanded production deployments. Railway operators can obtain a reliable, low-cost solution to continuously monitor their bridges and detect issues early to avoid disruptions.

This pilot demonstrates two significant benefits of the digital twin approach for structural health monitoring. First, it enables automated, continuous assessment of bridge integrity using only affordable wireless sensors. Second, the on-premises–cloud architecture provides a scalable implementation model that can be expanded to monitor thousands of bridges.

## 6. Conclusions

This pilot study demonstrated the capabilities of a digital twin solution for real-time monitoring of railway bridge structural integrity using low-cost accelerometers. The digital twin model was driven by vibration data streamed from the sensors to detect anomalies through machine learning algorithms to indicate potential damage. The hybrid edge–cloud architecture enabled an efficient and scalable implementation. The edge middleware handled high-speed data ingestion, preprocessing, and real-time FFT analysis, while the cloud provided storage, batch processing, and machine learning capabilities.

The digital twin’s machine learning algorithms were able to potentially detect and localize damage from changes in structural vibration patterns before visual inspection. This structural health monitoring approach based on a digitally twinned machine learning model provides an automated way to continuously assess bridge safety. It generates actionable insights on maintenance needs to prevent infrastructure failures and to avoid disasters.

For future work, we aim to extend this successful railway bridge demonstration to broader production deployments. The low cost and ease of implementation make the digital twin concept scalable to enable monitoring of all critical bridges across a transportation network. By detecting issues early, this system has the potential to dramatically improve infrastructure safety and prevent catastrophic failures through affordable, large-scale structural health evaluation.

While the digital twin and machine learning approach shows promise for monitoring other types of infrastructure as well, we plan to first improve the fidelity of the digital twin model using advanced physics simulations and expanded sensor data for the railway bridge use case. We will also research ways to better quantify the extent, localization, and progression of any damage detected.

One promising infrastructure application is in wind towers. These massive structures undergo important stresses and lack extensive instrumentation in the structure itself. A digital twin could simulate wind tower structural dynamics to pinpoint damage using limited sensor data. In this regard, the authors are exploring partnerships to develop digital twin monitoring systems for wind towers: both onshore and offshore. By detecting issues early, costly shutdowns and repairs could be avoided while ensuring these renewable energy assets operate safely.

While the current system architecture utilizes middleware for preprocessing and cloud machine learning, future work will shift more of the analytics onto intelligent sensors that provide energy harvesting and edge computing characteristics. The authors are developing custom sensors that can process FFT and vibration analysis directly on the sensor hardware using embedded machine learning chips. These sensors utilize e-peas [61] MPPT algorithms, state-of-the-art solar panels from Voltaic Systems [62], and Li-ion super capacitors for energy harvesting and storage. Edge Impulse will be used to process the data at the edge: progressively moving the middleware functionality from the fog to the edge. The sensors are based on ESP32 [63] technology and provide powerful edge computing capabilities at a low cost. This edge processing will reduce the data throughput burden on the middleware and cloud components. With smart sensors performing feature extraction on-chip, only the key vibration metrics will need to be transmitted instead of raw sensor streams. Such intelligent sensors would enable highly distributed analytics, with the cloud focus shifted to centralized data aggregation, model management, training, and decision making. The overall architecture would become more distributed but still retain cloud scale for global model building across bridges. Embedding intelligence directly into the sensors will eventually allow real-time embedded inference at the true edge, while cloud and on-premises resources will handle system-level coordination and big data analytics.

The core technologies proven here—low-cost IoT sensors, middleware processing, a digital twin, and machine learning—provide a framework to enable low-cost, data-driven structural health monitoring across infrastructure domains.

## Figures and Tables

**Table 1:** Descriptive data of the FFT registries.

	Principal_1	Principal_2	Principal_3
Mean	5.73	17.62	18.2
Median	5.62	20.02	7.57
Std. Deviation	0.17	20.26	25.84
Minimum	4.64	0	0
Maximum	6.84	100.83	119.87
Range	2.20	100.83	119.87

**Table 2:** Cluster centers and cases.

Cluster	Principal_1	Principal_2	Principal_3	Cases
ok_cluster_1	5.69	97.86	23.6	190
ok_cluster_2	5.68	14.39	71.21	659
ok_cluster_3	5.75	13.84	7.85	3474

**Table 3:** Descriptive cluster data.

	Cluster	Mean	Std. Deviation	Minimum	Maximum
principal_1	3	5.75	0.18	4.64	6.84
	2	5.67	0.11	5.62	6.35
	1	5.69	0.11	5.62	5.86
principal_2	3	13.93	11.17	0	102.83
	2	14.38	10.64	3.17	74.95
	1	97.86	6.86	73.49	100.83
principal_3	3	7.86	8.11	0	35.4
	2	71.18	23.11	50.29	119.87
	1	23.6	19.99	0	86.67

## Data Availability

Data are contained within the article.
